# Performance Assessment of a Multiplex Real-Time PCR Assay for Detection of Viruses Causing Respiratory Tract Infections

**DOI:** 10.3390/diagnostics14212350

**Published:** 2024-10-22

**Authors:** Pallavi Upadhyay, Fahida Surur, Vijay Singh

**Affiliations:** R&D Department, HealthTrackRx, Denton, TX 76207, USA; pallavi.upadhyay@healthtrackrx.com (P.U.); fahida.surur@healthtrackrx.com (F.S.)

**Keywords:** respiratory tract infections, respiratory viruses, SARS-CoV-2, RSV, influenza virus, multiplex RT-PCR, molecular diagnostics

## Abstract

Objectives: Following the COVID-19 pandemic, global epidemiological trends demonstrate a return to pre-pandemic levels of respiratory syncytial virus (RSV) and influenza (Flu) A/B viruses. For the appropriate clinical management of viral infections, reliable and timely diagnosis is crucial. The clinical presentation of these respiratory viral infections shows significant overlaps; thus, the syndromic diagnosis of these infections becomes challenging. The goal of this study was to compare the performance of three multiplex real-time PCR-based platforms for the detection of SARS-CoV-2, Flu A, Flu B, and RSV. Materials and Methods: A retrospective study was performed on 200 de-identified nasopharyngeal and oropharyngeal specimens. All samples were tested simultaneously on three PCR-based platforms for the detection of SARS-CoV-2, Flu A, Flu B, and RSV: HealthTrackRx’s real-time PCR Open Array^®^ respiratory panel, TrueMark™ SARS-CoV-2, Flu A, Flu B, RSV Select Panel, and BioFire^®^ RP2.1 Panel. The positive and negative predictive value of each test was evaluated at a 95% confidence interval. Results: Among the 200 tested samples, the TrueMark™ and OpenArray^®^ laboratory-developed tests (LDTs) showed a 100% concordance for the detection of SARS-CoV-2, Flu A, Flu B, and RSV. Overall agreement of 100% was observed for nasopharyngeal samples between the laboratory-developed tests and FDA-approved BioFire^®^ RP2.1 Panel. Diagnostic results for these four respiratory viruses, in clinical samples, between the LDTs and the FDA-approved comparator demonstrated full concordance. Conclusions: Respiratory viral infections represent one of the major global healthcare burdens. Consequently, the accurate detection and surveillance of these viruses are critical, particularly when these viruses are known to co-circulate. The excellent performance and full concordance of the LDTs, with the BioFire^®^ Respiratory RP2.1 panel, in detecting SARS-CoV-2, Flu A, Flu B, and RSV shows that these tests can be confidently implemented for the clinical testing of respiratory viral infections.

## 1. Introduction

Infectious diseases, particularly those affecting the respiratory system, not only pose significant threats to public health but also impose a substantial toll on economies worldwide. Influenza viruses, SARS-CoV-2, and respiratory syncytial virus (RSV) are among the most prominent viruses causing respiratory infections, resulting in widespread illness, hospitalization, and fatalities [1].

According to the Centers for Disease Prevention and Control (CDC), the economic burden of the influenza virus in the United States alone is substantial, with estimated annual costs ranging from USD 11.2 billion to USD 35.4 billion, attributed to direct medical expenses and indirect costs associated with lost productivity and absenteeism [2]. Regarding SARS-CoV-2 and the global pandemic it caused, the economic toll has been unmatched, with the cumulative burden in the United States surpassing USD 5.6 trillion as of January 2022. This astonishing figure encompasses healthcare expenses, economic disruptions, and the far-reaching consequences of the pandemic on business, employment, and social welfare programs [3]. Similarly, RSV imposes a significant burden on the healthcare systems; in particular, young children and older adults with comorbidities are more susceptible to RSV [4]. Compared with influenza and SARS-CoV-2, comprehensive data on the economic impact of RSV are limited; however, a recent systematic review indicates substantial healthcare costs associated with the direct cost burden of RSV hospitalizations at USD 1.3 billion for all adults [4].

The seasonality of respiratory viruses underwent significant changes during and after the COVID-19 pandemic. Strict non-pharmaceutical interventions such as lockdowns, mask mandates, social distancing, and travel restrictions were implemented to curb the spread of the virus. These measures inadvertently led to a decrease in the transmission of respiratory viruses such as influenza, RSV, and common coronaviruses. This decline was particularly noticeable during the 2020–2021 flu season, with historically low levels of influenza cases reported globally and suppressed RSV activity noted during this period [5].

However, as the pandemic-related restrictions on travel and congregation eased in 2021, a resurgence in cases of influenza virus and RSV was reported in the subsequent winter season [6]. In fact, the co-circulation of these three viruses in the United States led to an overwhelmed healthcare system in the winter of 2022–2023. This co-circulating phenomenon of the three viruses simultaneously within the population was coined as the “tripledemic” [7]. According to the CDC [8], flu and RSV incidences spiked during the fall–winter of 2023–2024 in a trend similar to what has been observed during the pre-pandemic years. These viruses thrive in colder temperatures and lower humidity, as well as in close indoor environments, where people congregate during the season [9]. The symptoms of these winter viral infections often depict influenza-like illness (ILI) and overlap with several other clinical presentations. In addition, the co-circulation of these viruses makes it difficult to distinguish the causative agent of respiratory illness [9].

For the last three years, ILI has been majorly dominated by SARS-CoV-2. With the COVID-19 pandemic-related public health emergency now declared over and given the re-emergence of common winter viruses (Flu A, Flu B, and RSV) and the similarities of clinical symptoms they present, multiplex real-time PCR testing remains crucial for distinguishing and identifying the causative agent of the respiratory illness [7]. According to the CDC’s latest guidelines [10], multiplex PCR testing is recommended for patients with respiratory illness (especially hospitalized, certain high-risk patients, and patients with comorbidities), enabling timely and accurate diagnosis and establishing control measures for infection spread. Undoubtedly, multiplex PCR tests are advantageous tools in the rapid detection of several pathogens that are associated with specific clinical syndromes in a single test [11], thus allowing healthcare providers to make timely diagnoses, treatment, and effective patient outcomes.

In this study, we clinically assessed the performance of a multiplex RT-PCR laboratory-developed test (LDT): the TrueMark™ SARS-CoV-2, Flu A, Flu B, RSV Select Panel, designed to detect and differentiate the four viruses in a single reaction. We further compared the performance of this test to two different multiplex PCR-based platforms, which detect SARS-CoV-2, Flu A, Flu B, and RSV in a single reaction: an LDT that is based on a TaqMan^®^ real-time OpenArray^®^ PCR platform and an FDA-approved in vitro diagnostic (IVD) test, the BioFire^®^ Respiratory 2.1 (RP2.1) Panel.

## 2. Materials and Methods

A total of 200 (141 nasopharyngeal and 59 oropharyngeal) de-identified samples were tested for the presence of SARS-CoV-2, Influenza A, Influenza B, and RSV. These specimens were residual samples obtained from routine testing of symptomatic individuals. These samples were analyzed and reported employing a real-time PCR molecular testing nano fluidic Open Array^®^ platform by HealthTrackRx Laboratory (Denton, TX, USA). The stored samples were used for testing in two panel PCR platforms: TrueMark™ SARS-CoV-2, Flu A, Flu B, RSV Select Panel and BioFire Respiratory Panel 2.1 (RP 2.1). The outcomes derived from multiplexed panels were assessed in terms of (i) determining positivity or negativity for individual analytes, (ii) evaluating the level of concordance among identical analytes, and (iii) analyzing performance by examining the correlation with cycle threshold (Ct) values when applicable.

Patient swabs were suspended in the PrimeStore^TM^ molecular transport medium (Longhorn Diagnostics, Bethesda, MD, USA), and nucleic acid isolation was performed using 200 µL of the sample following the manufacturer’s instructions using the MagMAX^TM^ viral/pathogen nucleic acid isolation kits on the automated KingFisher^TM^ Flex Purification System (ThermoFisher Scientific, Carlsbad, CA, USA).

### 2.1. Multiplex Expanded Panel Testing

For multiplex expanded panel testing, nucleic acid extraction was performed using Kingfisher Flex automated extraction system with MagMax^TM^ Viral/Pathogen II (MVP II) Nucleic Acid Isolation Kit (ThermoFisher Scientific, Carlsbad, CA, USA), as previously described [12]. Subsequent nucleic acid analysis was conducted using the QuantStudio^TM^ 12K Flex real-time PCR system, Gene Expression Open Array^®^ program (ThermoFisher Scientific, Carlsbad, CA, USA), targeting 32 viral and bacterial targets. Testing was executed at the HealthTrackRx laboratories. These include *Chlamydia trachomatis*, *Escherichia coli*, *Treponema pallidum*, *Staphylococcus aureus*, *Neisseria gonorrhoeae*, *Streptococcus agalactiae*, *Pseudomonas aeruginosa*, *Klebsiella (pneumoiae*, *oxytoca)*, *Chlamydia pneumoniae*, *Acinetobacter baumannii*, *Bacillus atrophaeus*, *Proteus (mirabilis*, *vulgaris)*, *Legionella pneumophila*, *Streptococcus pneumoniae Moraxella catarrhalis*, *Mycoplasma pneumoniae*, *Enterobacter (aerogenes*, *cloacae)*, *Fusobacterium (necrophorum*, *nucleatum)*, *Bordetella (pertussis*, *parapertussis*, *bronchiseptica)*, *Haemophilus influenzae*, *Streptococcus dysgalactiae*, *Streptococcus pyogenes*, *Serratia marcescens*, Respiratory syncytial virus A, Respiratory syncytial virus B, Enterovirus, Enterovirus D68Influenza A, Influenza B, Human metapneumovirus, Epstein–Barr virus, Adenovirus, SARS-CoV-2, Rhinovirus, Herpes simplex virus 1, Herpes simplex virus 2, Parainfluenza virus (1, 2, 3, 4), Coronavirus (229E, NL63), and Coronavirus (HKU1, OC43). A final volume of 2.5 µL of the sample was used for the OpenArray^TM^ reaction. The PCR cycling conditions were as follows: initial enzyme activation at 95 °C for 10 min, followed by 40 cycles of denaturation at 95 °C for 15 s and annealing/extension at 60 °C.

### 2.2. Multiplex Single Panel Testing

For multiplex single panel testing, remnant samples of 200 µL volume were subjected to testing using the TrueMark™ SARS-CoV-2, Flu A, Flu B, RSV Select Panel (ThermoFisher Scientific, Waltham, MA, USA) with the Kingfisher Flex automated extraction system. The TrueMark™ assay targeted specific genes for each pathogen, including open reading frame 1a, open reading frame 1b, and nucleocapsid (N) genes for SARS-CoV-2, PB1 and matrix (M) genes for Influenza A, matrix (M) gene and nonstructural (NS) gene for Influenza B, and nucleoprotein (NP), matrix (M), and L protein genes for RSV AB. Each reaction mixture contained 13 µL of extracted nucleic acid and 12 µL of one-step RT-PCR master mix (6.25 µL of TrueMark™ 1-Step Select Master Mix (No ROX), 1.25 µL TrueMark™ SARS-CoV-2, Flu A, Flu B, RSV Select Assay, and 4.5 µL of RNase-free water) at a final volume of 25 µL. Multiplex RT-PCR was performed and analyzed using the QuantStudio^TM^ 5 real-time PCR System (ThermoFisher Scientific, Carlsbad, CA, USA) as per the manufacturer’s instructions.

### 2.3. Biofire^®^ FilmArray^®^ Torch System Testing

A 300 µL specimen (nasopharyngeal and oropharyngeal swab) was tested on the Biofire^®^ FilmArray^®^ Torch System using BioFire Respiratory Panel 2.1 (RP 2.1) (bioMérieux, St. Louis, MO, USA) in adherence to the manufacturer’s instructions. The system targeted 18 viral and four bacterial targets, employing automated nucleic acid extraction, reverse transcription, nucleic acid amplification, and automated results analysis, with each target in a valid run reported as either ‘Detected’ or ‘Not Detected’. Each sample was processed individually, and work areas were cleaned following the manufacturer’s guidelines.

### 2.4. Statistical Analysis

Agreement between tests was represented as 2 × 2 contingency tables. Positive predictive agreement (PPA) and negative predictive agreement (NPA) were calculated with a 95% confidence interval and represented as a percentage value. Cycle threshold (Ct) values of the real-time PCR targets are presented as box and whisker plots to represent the range of Ct values demonstrated by the samples. Student’s *t*-test was performed to compare the two PCR LDTs for each viral target. A *p*-value of less than 0.05 was considered to be a statistically significant difference. All statistical analyses were performed using R version 4.3.2 (R Foundation for Statistical Computing, Vienna, Austria).

## 3. Results

In this retrospective analysis, we evaluated the performance of two laboratory-developed tests—TrueMark™ SARS-CoV-2, Flu A, Flu B, RSV Select Panel (TM) and TaqMan^®^ real-time OpenArray^®^ PCR platform (OA)—and an FDA-approved diagnostic test, the BioFire^®^ Respiratory 2.1 Panel (RP2.1), for the detection of SARS-CoV-2, Influenza A, Influenza B, and RSV in 200 upper respiratory swab samples collected from individuals presenting with influenza-like illness. The respiratory samples tested in this cohort comprised nasopharyngeal (*n* = 141) and oropharyngeal (*n* = 59) swabs collected within the continental United States during the 2023/2024 flu season.

TM and OA tests included in the comparison study showed 100% concordance for the detection of SARS-CoV-2, Influenza A, Influenza B, and RSV (Table 1). The positive percent agreement achieved for all four targets for both the LDTs compared was 100% (95% CI, 91.24–100.00%), and the negative percent agreement was also 100% for all four targets (95% CI, 97.66–100.00%). The RP2.1 test is IVD-approved for nasopharyngeal samples only, and the PPA of the test for the four viral respiratory targets, compared with the OA and TM LDTs, was 100% (95% CI, 96.87–100.00%). Similarly, the NPA for RP2.1 was 100% (95% CI, 86.28–100.00%) (Table 2). No co-infections between the four viruses were observed in this sample cohort.

**Figure 1:**
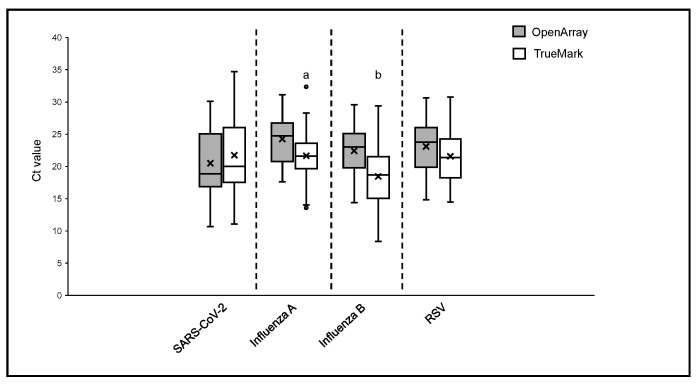
Ct values comparison between OpenArray^®^ and TrueMark™ Select Panel detected for the detection of SARS-CoV-2, Flu A, Flu B, and RSV. Alphabets represent statistically significant differences between the two PCR tests (*p* < 0.05) in a *t*-test analysis.

**Figure 2:**
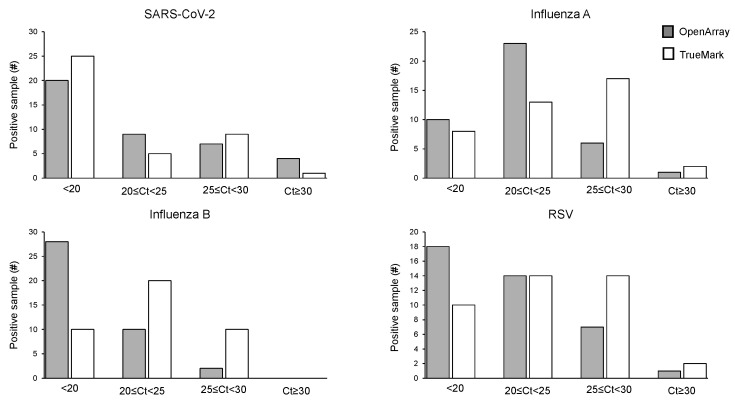
Distribution of Ct values for the positive cohort on OpenArray^®^ and TrueMark^TM^-based multiplex PCR laboratory-developed tests for the detection of SARS-CoV-2, Influenza A, Influenza B, and RSV.

The positive sample cohorts for SARS-CoV-2, Influenza A, and RSV viruses spanned the dynamic range of the tests evaluated, with at least 5% of samples showing high, medium and low viral loads as indicated by Ct values of the two LDT panels, which report them (Figure 1). Samples positive for Influenza B showed high and medium viral loads, with no samples included with Ct > 30 (Figure 2). When compared to the OA test, the Ct values obtained using the TM test for Influenza A, Influenza B, and RSV were, on average, lower, with the median difference observed varying from 1.55 Ct for RSV to 3.77 Ct for Influenza B (Figure 1). The difference in the Ct values was statistically significant for Influenza A (*p* = 0.0027) and Influenza B (*p* = 0.0001) on the TM test, which was significantly lower than the OA test. In contrast, the Ct values for SARS-CoV-2 were lower on the OA plate compared with the TM test, with a median difference of 1.3 Ct (Figure 1). As the RP2.1 Panel does not report Ct values, it was not included in the analysis.

**Figure 3:**
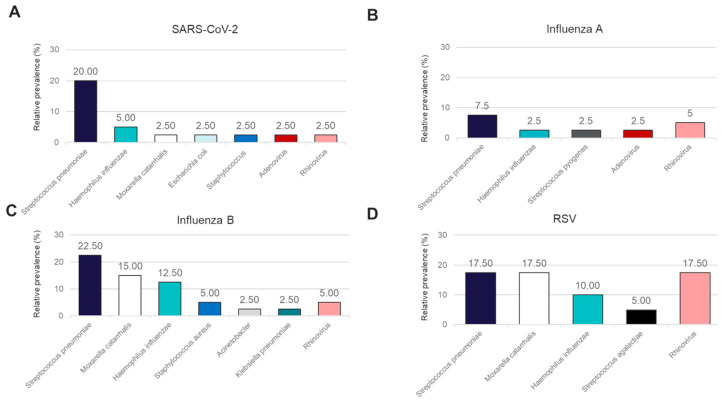
Relative prevalence of coinfecting organisms identified using the OpenArray^®^ PCR platform among specimens that tested positive for (**A**) SARS-CoV-2, (**B**) Influenza A, (**C**) Influenza B, or (**D**) RSV.

In addition to performance for the detection of the four major causes of viral respiratory illness, we also analyzed the co-infections detected in this sample cohort using the OA LDT and the RP2.1 Panel. While the BioFire^®^ Respiratory 2.1 (RP2.1) Panel mainly detects viral pathogens associated with respiratory illnesses and four bacterial causes of atypical pneumonia, the OA LDT covers, in addition, a broader range of bacterial pathogens involved in respiratory tract infections. Both panels showed 100% concordance in the detection of viral co-infections for Rhinovirus and Adenovirus (Figure 3A–D). Rhinovirus co-infections were detectable in samples positive for SARS-CoV-2, Influenza A, Influenza B, and RSV, while Adenovirus was detected only in conjunction with SARS-CoV-2 and Influenza A. A high rate of Rhinovirus co-infection was observed for RSV (17.5%) (Figure 3D). Bacterial co-infections were detectable in this sample cohort and could only be detected using the OA LDT (Figure 3A–D). The most frequent bacterial co-infections observed with all four primary viral pathogens included *Streptococcus pneumoniae* (range 7.5–22.5%) and *Haemophilus influenzae* (range 2.5–12.5%) (Figure 3A–D). *Moraxella catarrhalis* co-infections were not detected in the Influenza A-positive sample cohort, whereas positivity rates for the other three groups varied from 2.5% in SARS-CoV-2-positive samples to 17.5% in RSV-positive samples (Figure 3A–D).

## 4. Discussion

Respiratory tract infections caused by viral pathogens constitute a significant economic burden on healthcare systems globally. A propensity-matched analysis of the National Inpatient Sample database between January and December 2020 demonstrated that COVID-19-associated acute respiratory distress syndrome (ARDS), in comparison to influenza-associated ARDS, had significantly higher rates of mortality and requirement for mechanical ventilation [13]. Prior to the recent introduction of the RSV vaccine for adults aged 60 years and older, a prospective multi-center clinical trial showed that RSV disease severity was similar to COVID-19 or influenza in patients not vaccinated against these viruses, whereas the disease severity was significantly higher among vaccinated patients [14].

The syndromic nature of viral respiratory tract infections exacerbates the situation with respect to the diagnosis of the correct etiological agent, as there is a significant overlap in the symptoms presented by the patient. Influenza A, Influenza B, and RSV are some of the most common viral pathogens responsible for lower respiratory tract infections in both children and adults [15,16]. The outbreak of the COVID-19 pandemic further complicated this scenario, with the SARS-CoV-2 virus becoming a leading contributor to an increase in all-cause lower respiratory tract infections during the years 2020–2022 when compared to the pre-pandemic period [17]. As newer variants of the SARS-CoV-2 virus emerged, the symptomology associated with the infection appeared progressively similar to that of other respiratory viral infections, especially Influenza and RSV. This has rendered identification and surveillance based on syndrome alone both difficult and ineffective, making it necessary that multiple pathogens can be tested at the same time [18].

In this study, we compared the clinical performance of two laboratory-developed multiplex PCR tests to the FDA-approved BioFire FilmArray PCR (RP2.1) tests for the detection of four viral pathogens (SARS-CoV-2, Influenza A, Influenza B, and RSV) that cause respiratory tract infections. With the OA LDT used as the reference, both BioFire RP2.1 and TM LDT showed full concordance with 100% PPA and NPA (Table 1 and Table 2).

Information on pathogen load is important for making clinical decisions regarding effective infection control and management. The cycle threshold (Ct) value generated during PCR is a good indicator of the amount of target pathogen present in a sample. However, reporting of Ct values for positive pathogen detection as an indicator of clinical outcomes has been shown to be effective in some but inconclusive in other cases for respiratory [19] and gastrointestinal infections [20]. These inconsistencies have been attributed to a lack of standardization in generating and utilizing Ct values for infection management. The two LDTs used in this study utilize similar PCR chemistry, but the actual reaction is performed on different instruments with varying sample throughput capacities. The TM LDT can be performed on 96- and 384-well plastic PCR plates, whereas the OA LDT utilizes custom nanofluidic chips that can test up to 192 samples in a single run. Oropharyngeal and nasopharyngeal clinical samples tested for the presence of SARS-CoV-2, Influenza A, Influenza B, and RSV at varying pathogen loads demonstrated comparable Ct values, with statistically significant differences observed for Influenza A and Influenza B (Figure 1). Differences in Ct values observed for the same sample when tested on different platforms may be a result of assay optimization and real-time PCR efficiency, which can vary between the tests. Respiratory viral infections are subject to seasonal variations, with a clear peak in infection positivity rates during the fall and winter months. This seasonality is also reflected in the specimen testing volume encountered by diagnostic labs [21]. Our results show the feasibility of using different PCR-based tests for the detection of respiratory viral pathogens that can dynamically adjust to the sample volume throughput of a laboratory without compromising test performance or turnaround time to results.

The FilmArray system from BioFire is an FDA-approved multiplex PCR test for the detection of respiratory viral pathogens from nasopharyngeal swabs. In comparison to other clinical methods for the detection of respiratory viral pathogens, the clinical utility of the FilmArray panels has been shown previously for various healthcare scenarios, including neonates [22], children [23], immunocompromised adult patients [24], and non-specific settings like the emergency department [25]. In a retrospective analysis of ICU-admitted patients with suspected pneumonia, concurrent testing with culture method and FilmArray pneumonia panels showed early and appropriate antimicrobial treatment in almost 90% of patients with a positive result on the multiplex PCR test [26]. Recently published results from a randomized clinical trial demonstrated that PCR test, utilizing the BioFire FilmArray Pneumonia panel, resulted in targeted and rapid microbial treatment in patients displaying symptoms of community-acquired pneumonia [27]. In the present study, our two LDTs demonstrated full concordance with the FilmArray RP2.1 panel with an overall agreement of 100% (Table 2). Although the focus of this study was the detection of SARS-CoV-2, Influenza A, Influenza B, and RSV, the IVD comparator panel also detects other relevant respiratory viral pathogens and atypical bacterial respiratory pathogens. The OA LDT panel has been designed to detect the presence of a wide range of viral and bacterial pathogens in the respiratory tract. In addition to the four viral pathogens under observation, the OA LDT co-detected the presence of Rhinovirus and Adenovirus in 10 nasopharyngeal samples. The Biofire IVD and OA LDT displayed 100% concordance in the detection of these additional pathogens. Co-detection of other bacterial pathogens in the tested samples (Figure 3) did not interfere with the detection of the viral pathogens.

Rapid diagnostic tests have been shown to reduce the length of hospital stay and overall cost to the system when used for respiratory viral infections [28]. For the diagnosis of respiratory viral infections, the retrospective analysis of clinical trial data has demonstrated the utility of multiplex PCR in reducing the time to results, length of hospital stays, and correct antiviral therapy, especially during the COVID-19 pandemic [29], but the utility of any new test under various clinical contexts still needs to be established. These include the impact on clinical utility in different patient settings, economic outcomes, clinical management decisions, and appropriate antimicrobial and antiviral therapy [30]. A limitation of the present study is that we established the clinical validity of the LDTs in comparison to an FDA-approved comparator test on archived respiratory samples. Although an overall agreement of 100% with the BioFire RP2.1 panel in detecting the four respiratory viral pathogens is a good indicator of the clinical utility of the LDTs, further research is needed to establish the same for different patient populations and use case scenarios.

## 5. Conclusions

Our results demonstrate the feasibility of confidently employing multiplex PCR LDTs for the diagnosis of SARS-CoV-2, Influenza A, Influenza B, and RSV in clinical settings. As compared to the BioFire IVD test, the LDTs have much higher throughput and can handle increased sample volume with similar sensitivity and specificity for the viral targets in diverse sample types, as well as the possibility of reporting semi-quantitative results. In addition, the LDTs are more amenable to workflow automation, allowing for increased efficiency within a clinical laboratory setting.

## Figures and Tables

**Table 1:** Concordance between the TrueMark^TM^ and OpenArray^®^ multiplex PCR tests for the detection of SARS-CoV-2, Influenza A, Influenza B, and RSV in nasopharyngeal and oropharyngeal clinical samples.

TrueMArk^TM^ SARS-CoV-2, FluA, FluB, RSV Panel		OpenArray^®^ SARS-CoV-2, FluA, FluB, RSV Panel							
		**SARS-CoV-2**		**Influenza A**		**Influenza B**		**RSV**	
		**Positive**	**Negative**	**Positive**	**Negative**	**Positive**	**Negative**	**Positive**	**Negative**
	**Positive**	40	0	40	0	40	0	40	0
	**Negative**	0	160	0	160	0	160	0	160
	PPA	100% (91.24% to 100%)		100% (91.24% to 100%)		100% (91.24% to 100%)		100% (91.24% to 100%)	
	NPA	100% (97.66% to 100%)		100% (97.66% to 100%)		100% (97.66% to 100%)		100% (97.66% to 100%)	

**Table 2:** Concordance between the TrueMark^TM^ and OpenArray^®^ multiplex PCR tests and BioFire^®^ Respiratory Panel 2.1 (RP2.1) for the detection of SARS-CoV-2, Influenza A, Influenza B, and RSV in nasopharyngeal clinical samples.

TrueMark^TM^/OpenArray^®^ SARS-CoV-2, FluA, FluB, RSV Panel		BioFire^®^ Respiratory 2.1 (RP2.1) Panel							
		**SARS-CoV-2**		**Influenza A**		**Influenza B**		**RSV**	
		**Positive**	**Negative**	**Positive**	**Negative**	**Positive**	**Negative**	**Positive**	**Negative**
	**Positive**	24	0	30	0	30	0	25	0
	**Negative**	0	117	0	112	0	112	0	109
	PPA	100% (85.75% to 100%)		100% (88.43% to 100%)		100% (88.43% to 100%)		100% (86.28% to 100%)	
	NPA	100% (96.90% to 100%)		100% (96.76% to 100%)		100% (96.76% to 100%)		100% (96.76% to 100%)	

## Data Availability

The data presented in this study are available upon reasonable request from the corresponding author V.S.
